# Elevated tumour interleukin-1*β* is associated with systemic inflammation: a marker of reduced survival in gastro-oesophageal cancer

**DOI:** 10.1038/sj.bjc.6603446

**Published:** 2006-11-07

**Authors:** D A C Deans, S J Wigmore, H Gilmour, S Paterson-Brown, J A Ross, K C H Fearon

**Affiliations:** 1 Tissue Injury and Repair Group, Department of Clinical and Surgical Sciences, MRC Centre for Inflammation Research, The Chancellor's Building, Edinburgh University, 49 Little France Crescent, Edinburgh EH16 4SB, UK

**Keywords:** inflammation, cytokines, real-time PCR

## Abstract

Systemic inflammation is associated with adverse prognosis cancer but its aetiology remains unclear. We investigated the expression of proinflammatory cytokines within normal mucosa from healthy controls and tumour tissue in cancer patients and related these levels with markers of systemic inflammation and with the presence of a tumour inflammatory infiltrate. Tissue was collected from 56 patients with gastro-oesophageal cancer and from 12 healthy controls. Tissue cytokine mRNA concentrations were measured by real-time PCR and tissue protein concentrations by cytometric bead array. The degree of chronic inflammatory cell infiltrate was recorded. Serum cytokine and acute phase protein concentrations (including C-reactive protein (CRP)) were measured by enzyme-linked immunosorbent assay. Proinflammatory cytokines were significantly overexpressed (interleukin (IL)-1*β*, IL-6, IL-8 and tumour necrosis factor-*α*) both at mRNA and protein levels in the cancer specimens compared with mucosa from controls. Interleukin-1*β* was expressed in greatest (10–100-fold) concentration and protein levels correlated significantly with systemic inflammation (CRP) (*P*=0.05, *r*=0.31). A chronic inflammatory infiltrate was observed in 75% of the cancer specimens and was associated with systemic inflammation (CRP: *P*=0.01). However, the presence of chronic inflammation *per se* was not associated with altered cytokine expression within the tumour. Both a chronic inflammatory infiltrate and systemic inflammation (CRP) were associated with reduced survival (*P*=0.05 and *P*=0.03, respectively). Tumour chronic inflammatory infiltrate and tumour tissue IL-1*β* overexpression are potential independent factors influencing systemic inflammation in oesophagogastric cancer patients.

Systemic inflammation has been found in association with the majority of advanced solid epithelial malignancies and at the time of diagnosis up to 50% of patients may have an elevated acute phase protein response (APPR) (Falconer *et al*, 1995). The presence of an APPR has been associated with weight loss, the presence of hypermetabolism and anorexia, extent of disease, the development of recurrence in advanced cancer, and adverse prognosis (independent of stage of disease) (Rashid *et al*, 1982; Kodama *et al*, 1999; McMillan *et al*, 2001, 2003; Nozoe *et al*, 2001; Forrest *et al*, 2003). In patients with gastric cancer, the presence of systemic inflammation has been associated with a markedly reduced median survival (9 *vs* 53 weeks, *P*<0.001) (Rashid *et al*, 1982). Similarly, a study from Japan has identified a shortened survival in oesophageal cancer patients with an elevated serum C-reactive protein (CRP) at the time of diagnosis (Nozoe *et al*, 2001). More recently, a group from the UK has identified elevated serum CRP and reduced serum albumin concentrations as independent prognostic indicators among patients with inoperable gastro-oesophageal cancer (Crumley *et al*, 2006). The systemic inflammatory response is highly complex and is modulated, in part, by the interaction of pro- and anti-inflammatory cytokines. However, the precise origin of systemic inflammation among cancer patients remains obscure.

Human cancer cell lines have been shown to produce proinflammatory cytokines (Gelin *et al*, 1991; Strassmann *et al*, 1993a, 1993b; Wigmore *et al*, 2002). However, such proinflammatory cytokines are not reliably detected in the circulation and probably act locally to promote inflammation and activate host inflammatory cells (e.g. peripheral blood mononuclear cells: PBMCs) passing through the tumour (Falconer *et al*, 1994; O'Riordain *et al*, 1999). Such cells can re-enter the circulation and release cytokines at distant target organs (e.g. the liver). More recently, Martignoni *et al* (2005) have suggested that interleukin (IL)-6 overexpression in pancreatic cancer patients is related to the ability of certain IL-6 producing tumours to sensitise PBMC and induce IL-6 expression in PBMCs. The main cytokines influencing the APPR in humans are thought to include IL-6, IL-1*β*, and tumour necrosis factor-*α* (TNF-*α*) (O'Riordain *et al*, 1999). Interleukin-6 is the main inducer of the APPR in human hepatocytes and both IL-1*β* and TNF-*α* are capable of inducing IL-6 production from both tumour and host cells (Strassmann *et al*, 1993a, 1993b). In cancer patients, the rates of production of IL-6 from isolated PBMCs can be linked to markers of systemic inflammation such as CRP (O'Riordain *et al*, 1999). The presence of such an acute phase reaction may then be used as an indirect marker of proinflammatory cytokine activity (IL-1*β*, IL-6, and TNF-*α*).

The source of the proinflammatory stimulus in advanced cancer remains unclear. It has been hypothesised that in patients with cancer, either the tumour cells or the host cells or a combination of the two are responsible for the production of the proinflammatory cytokines that induce the APPR. With a view to modulation of systemic inflammation in cancer, we hypothesise that dominant cytokines within tumour tissue drive the systemic inflammatory response and that these might be considered as targets for specific therapy. To investigate the role of tumour tissue in the genesis of systemic inflammation in cancer patients, we measured cytokine (IL-1*β*, IL-6, IL-8, and TNF-*α*) mRNA and protein concentrations in tumour tissue collected from patients with gastro-oesophageal cancer and tissue from healthy controls and related these measurements to systemic concentrations of cytokines and acute phase proteins (APPs). We also investigated the significance of a chronic inflammatory cellular infiltrate within these tissues and related these findings to tissue cytokine concentrations and to clinical outcome.

## PATIENTS AND METHODS

### Study patients

Patients diagnosed with gastric or oesophageal cancer within the Lothian and Borders regions between June 2002 and March 2004 were eligible for inclusion into the study. Patients were recruited at the time of diagnosis and all subjects provided written informed consent and the study received ethical permission from the Lothian Research Ethics Committee. All patients who had surgery were eligible and were studied. No patients were excluded or refused consent. Patients not suitable for surgical resection (advanced disease stage or comorbidity) were excluded from the study. Patients were staged according to the International Union Against Cancer (UICC), and final histopathological stage (pTNM) was used in all cases (Sobin and Wittekind, 2003). Tumours located around the oesophago–gastric junction were classified according to Siewert and those classified as types I and II were staged as oesophageal tumours and type III as gastric cancers (Siewert and Stein, 1998). All clinical and pathological information was collected prospectively, including documentation of the use of nonsteroidal anti-inflammatory drugs (NSAIDs) and any other therapeutic agents that may influence the inflammatory response.

### Determination of serum APP and cytokine concentrations

A random blood was collected from patients at the time of diagnosis and before any therapeutic intervention. All patients were free from infection at the time of blood collection. Samples were collected simultaneously from 22 healthy controls for comparison. Serum was obtained by collecting whole blood into lithium-heparinised tubes and centrifuging at 2000 r.p.m. for 10 min at 10°C (Mistral 3000i, Thermo Life Sciences, Basingstoke, UK). Aliquots were stored at −80°C until batch analysis.

C-reactive protein was determined using an immunoturbidimetric assay (Abbott TDX, Abbott Laboratories, Maidenhead, UK). A level above 10 mg l^−1^ defined the presence of an APPR. Serum albumin concentrations were measured by an automated bromocresol green dye-binding technique. The remaining APPs were determined by sandwich enzyme-linked immunosorbent assay as described previously (Wigmore *et al*, 2002). Briefly, 96-well plates were coated with 100 *ì*l primary antibody (concentration 10 mg l^−1^) and incubated overnight at 4°C (Dako, Ely, UK). The plates were washed with 0.1% Tween and diluted sera (100 *μ*l) was added to the coated wells and incubated at room temperature for 2 h. Plates were washed as before and a secondary antibody conjugated with peroxidase was added to each well and incubated for 1 h (Dako, Ely, UK). The substrate used was OPD (Dako, Ely, UK) and the reaction was stopped with 0.5 M sulphuric acid. Plates were read at 490 nm using a Dynatech MR5000 automated plate reader. Standard curves were generated using standard APPs supplied by the manufacturer (Dako, Ely, UK).

Serum cytokines were analysed with module kits and performed according to the manufacturers instructions (Caltag, Bender MedSystems, Towcester, UK). The lower limit of sensitivity for each assay was; <1 pg ml^−1^ IL-1*β*, 1.4 pg ml^−1^ IL-6, 11 pg ml^−1^ IL-8, 0.8 pg ml^−1^ IL-10, and 5.8 pg ml^−1^ TNF soluble receptor (sTNF-R).

### Tissue cytokine mRNA and protein measurement

#### Tissue collection

Tissue was obtained from 56 patients at the time of surgical resection. A representative sample of tumour tissue was collected from each patient and tissues were snap frozen in liquid nitrogen before storage at −80°C until further analysis. An additional 12 patients were recruited as healthy controls. These patients underwent endoscopy as an elective procedure for investigation of dyspeptic-type symptoms. In all instances, the result of the procedure was normal, including both macroscopic and microscopic assessment. Mucosal tissue (seven oesophageal and five gastric) samples were collected from these patients with biopsy forceps at the time of endoscopy. All control subjects were considered healthy without established comorbidity or taking regular medications.

### Quantitative reverse transcription–polymerase chain reaction (Q-RT–PCR)

#### RNA isolation and RT

Total RNA was isolated from tissue samples using the RNeasy kit (Qiagen Inc., Crawley, UK). RNA quality and integrity was assessed using an Agilent 2100 bioanalyser (Agilent Technologies Ltd, Chesire, UK) in five randomly selected samples. For the remaining samples, purity and concentration were determined using spectrophotometry (Ultrospec 2000, Pharmacia Biotech, Bucks, UK). Reverse transcription was performed using 1 *μ*g of total RNA following DNase treatment (Qiagen Inc., UK). All RNA samples were checked for genomic DNA contamination before RT using conventional RT–PCR. Two microlitres of total RNA was mixed with 1 *μ*l MgCl (25 mM), 2.5 *μ*l 10 × *Taq* DNA polymerase buffer with added MgCl, 2.5 *μ*l dNTP (10 mM), 5 *μ*l forward and reverse primers (10 *μ*M), 11 *μ*l DEPC-treated water and 1 *μ*l *Taq* DNA polymerase (5 U *μ*l^−1^) (all reagents Promega, Southampton, UK). Primers for cytochrome *b* were used to detect DNA contamination. The forward primer sequence was GGTTCTGGAATAAGAATATAGG and the reverse primer sequence GACAACACAGTAAGAACCAGG, giving a product of 367 bp if contamination was present.

Reverse transcription was performed once DNA contamination had been excluded. The reaction mixture included the RNA (1 *μ*g in 10 *μ*l DEPC-treated water), 4 *μ*l MgCl (25 mM), 2 *μ*l 10 × reverse transcriptase buffer, 2 *μ*l dNTPs (10 mM), 1 *μ*l random hexamers (500 *μ*g ml^−1^), 1.5 *μ*l AMV reverse transcriptase (10 U *μ*l^−1^), and 0.5 *μ*l recombinant RNase inhibitor (40 U *μ*l^−1^) (all reagents Promega, Southampton, UK). Reverse transcription was performed at 42°C for 60 min followed by 95°C for 5 min.

#### Real-time PCR

Quantitative PCR was performed using the ABI PRISM 770 real-time Sequence Detection System (Applied Biosystems, Warrington, UK). Reactions were performed in 50 *μ*l total volume, consisting of; 25 *μ*l Taqman universal PCR master-mix (UNG × 2), 14 *μ*l primer/probe mix, 2.5 *μ*l ribosomal 18S primer/probe mix (all reagents Applied Biosystems, UK), 3.5 *μ*l DEPC-treated water, and 5 *μ*l cDNA. Each sample was analysed in duplicate. The reaction conditions were 2 min at 50°C, 10 min at 95°C, and 40 cycles with 15 s at 95°C and 1 min at 60°C. Genes studied included IL-1*β*, IL-6, IL-8, and TNF-*α*. The primers and probes were designed by Applied Biosystems, UK.

Quantification of gene expression was calculated using the comparative (ΔΔ*C*_T_) method, where samples were compared with the positive control (Bustin, 2000). The level of gene expression within each sample was adjusted to an internal control (human ribosomal 18S) before expression was calculated as a percentage of the level of gene expression by the control sample. Samples that generated cycle numbers above 23 for the endogenous control (18S) were discarded and the samples were repeated.

#### Positive control

Whole blood was collected from healthy donors and the white cells were isolated using histopaque (Sigma, Dorset, UK). The cells were cultured in lipo-polysaccharide (Sigma, Dorset, UK) for 48 h before isolation of the RNA. Total RNA was reverse transcribed as described above. Each real-time reaction used an aliquot from the stock solution of cDNA as a positive control.

#### Extraction of tissue protein

Tissue lysates were prepared by homogenising 50 mg of tissue in 400 *μ*l tissue homogenising buffer (0.4 ml 500 mM Tris, 0.2 ml 100 mM ATP, 1 ml 50 mM MgCl_2_, 10 *μ*l dithiothreitol, 1 × protease inhibitor, 8.4 ml water – Sigma, Dorset, UK). Samples were heated to 95°C for 5 min before centrifuging at 13 000 r.p.m. for 30 min. Protein concentration of the supernatants was determined by the Bradford method (Bio-Rad, Hemel Hempstead, UK) (Bradford, 1976). Samples were stored at −80°C until analysis.

### Determination of tissue cytokine concentrations

Cytokine protein concentrations were determined using the Cytometric Bead Array System according to manufacturer's instructions (Human Inflammation Kit, BD Biosciences, Oxford, UK). This kit allows the measurement of cytokines IL-1*β*, IL-6, IL-8, IL-10, IL-12p70, and TNF-*α*. Briefly, 50 *μ*l of tissue extract was added to the reaction mix containing antibody-coated microbeads and incubated at room temperature for 3 h. Cytokine concentrations were determined by flow cytometry (BD FACScan, Oxford, UK). Results were calculated to take into account the total protein concentration of the tissue lysate and are expressed as pg mg^−1^ of total protein. Intra-assay variability ranged between 2 and 10% and interassay variability was 4–15%.

### Histological analysis

**Figure 1:**
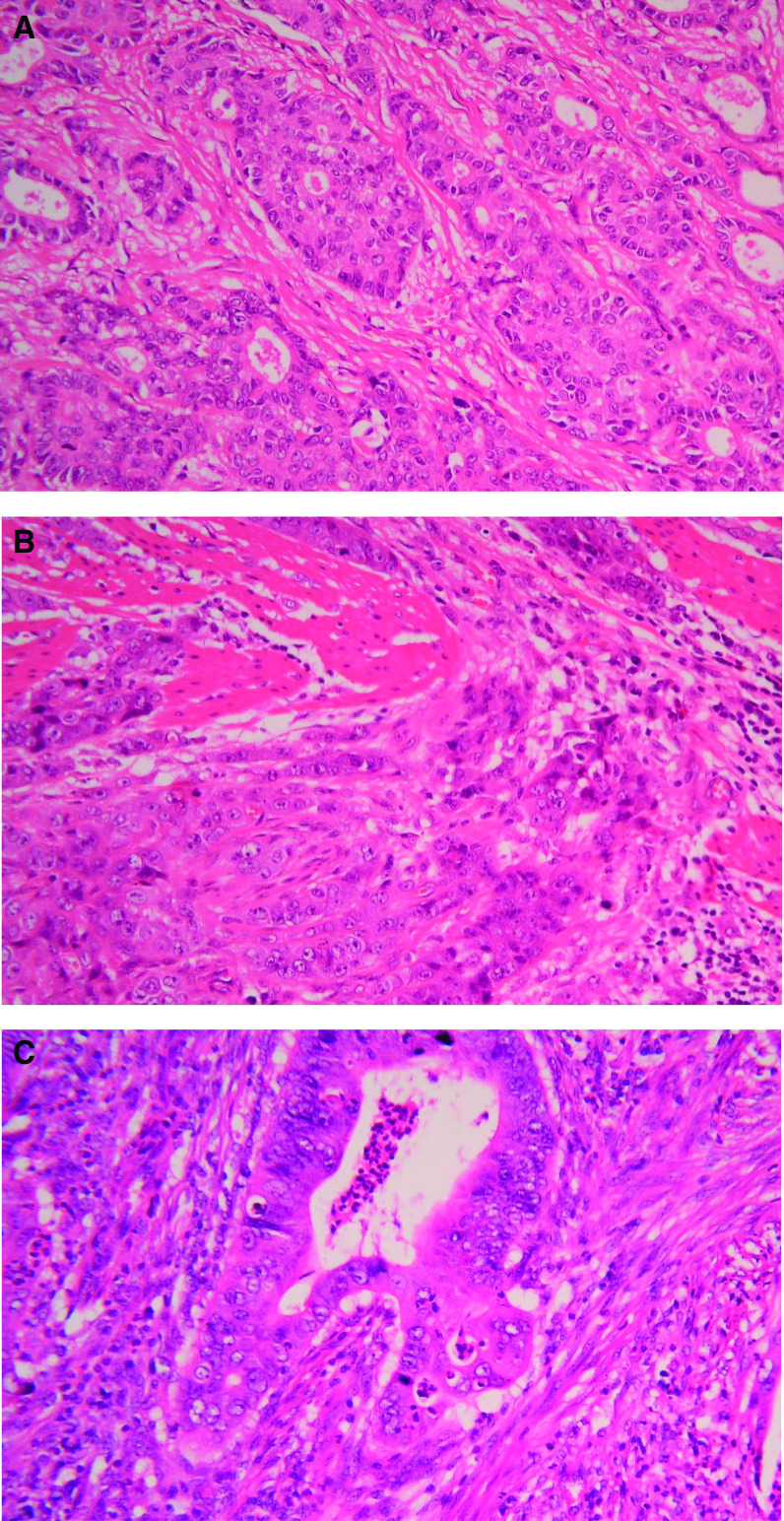
Representative photomicrographs taken from three patients with poorly differentiated adenocarcinoma of the oesophagus. Patient (**A**) demonstrates minimal/no inflammatory cell reaction. Patient (**B**) has a patchy chronic inflammatory cell infiltrate. Patient (**C**) shows a diffuse chronic inflammatory cellular infiltrate present throughout the tumour. Sections of tumour tissue were fixed with formalin and stained with haematoxylin and eosin (magnification × 100).

Representative sections of tumour tissue were fixed with formalin and stained with haematoxylin and eosin. A single Consultant pathologist (HG) reviewed all the tissue sections and the extent of a chronic inflammatory cellular infiltrate was recorded. Sections were classified as either diffuse scanty (occasional) chronic inflammatory cells present, focal lymphoid aggregates only, diffuse chronic inflammatory cellular infiltrate present throughout the tissue, or patchy chronic inflammatory cells present (Figure 1). HG was blinded to the clinical data, serum APP/cytokine concentrations, and tissue cytokine concentrations relating to each patient.

### Statistical analysis

Comparisons between groups of continuous variables were made by the Mann–Whitney *U*-test. Categorical variables were compared by Fisher's exact test. Correlations between continuous variables were assessed by Spearman's rank correlation coefficient. Survival between groups was analysed by the log-rank test and Cox's proportional hazards model. A *P*-value ⩽0.05 was considered statistically significant.

## RESULTS

### Study patients

Patient demographics are shown in Table 1. Subgroup analysis confirmed no significant differences in either tissue mRNA or protein levels between patients who received preoperative chemotherapy and those who did not (data not shown). Similarly, there were no differences in tissue mRNA or protein levels or serum cytokine or APP levels among those patients taking NSAIDs or any other therapeutic agents that may modify the inflammatory response (data not shown). Therefore, all patients were included as a single group for analysis.

### Serum cytokine and APP concentrations

**Figure 2:**
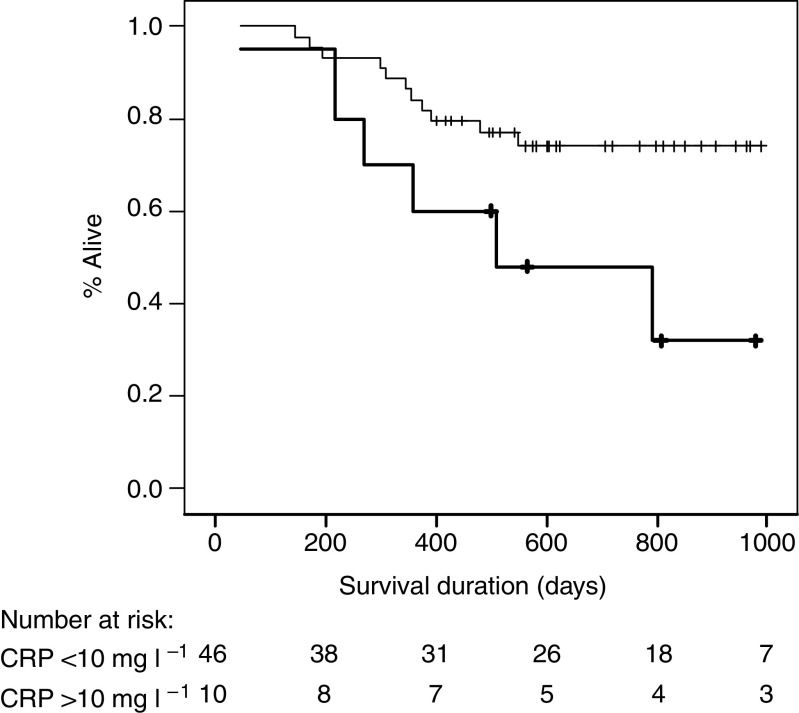
Kaplan–Meier survival plot presented by serum CRP concentration. Heavy line CRP >10 mg l^−1^ (median survival 509 days) *vs* light line CRP <10 mg l^−1^ (median survival >900 days); *P*=0.031, log-rank test.

Serum APP concentrations for the study patients and healthy controls are shown in Table 2. The patient group had significantly elevated concentrations of positive APPs compared with the control population; CRP (*P*<0.001, Mann–Whitney *U*-test), haptoglobin (*P*<0.001), and *α*1-antichymotrypsin (*P*<0.001). There was no difference in concentrations of the negative acute phase reactants; albumin (*P*=0.242) or transferrin (*P*=0.346). Ten (18%) patients had a serum CRP concentration >10 mg l^−1^, which was associated with reduced survival duration (*P*=0.031, log-rank test) (Figure 2). C-reactive protein concentration remained an independent prognostic indicator on multivariate analysis when analysed with stage, age, sex, and grade (*P*=0.048, hazard ratio 2.7 (1.1–7.3 95% CI); Cox's proportional hazards model).

Serum cytokine concentrations were similar between the healthy controls and cancer patients (Table 2). Serum cytokine concentrations did not correlate with serum APP concentrations (linear regression, data not shown) and patients with CRP levels greater than 10 mg l^−1^ did not have significantly elevated serum cytokine concentrations.

### Tissue cytokine mRNA and protein concentrations

**Figure 3:**
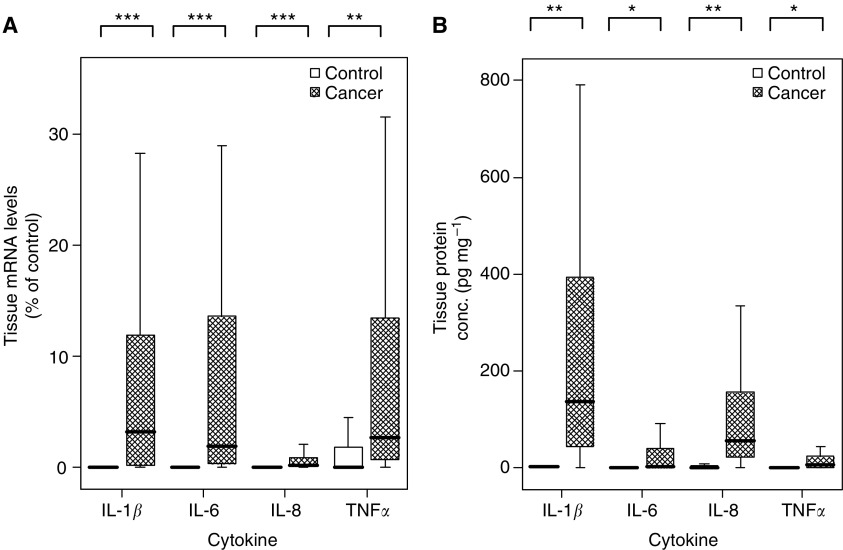
Comparison of cytokine levels of (**A**) mRNA and (**B**) protein between tissue from healthy controls and tumour tissue from patients with gastro-oesophageal cancer. The lines represent the median value, bars=interquartile range, error bars=extreme values. IL-1*β*=interleukin-1*β*, TNF-*α*=tumour necrosis factor-*α*. ^*^P<0.05, ^**^P<0.01, ^***^P<0.001 (Mann–Whitney *U*-test).

Interleukin-6 and IL-8 mRNA were not measurable in any of the gastro-oesophageal mucosa samples collected from healthy controls and IL-1*β* and TNF-*α* were only detectable at very low concentrations (Figure 3A). In contrast, mRNA for IL-1*β*, IL-6, IL-8, and TNF-*α* were detected in tumour tissue at significantly elevated concentrations: IL-1*β P*<0.001; IL-6 *P*<0.001; IL-8 *P*<0.001; TNF-*α P*=0.006 (see Figure 3A).

Similarly, IL-6 protein was not detected in mucosal tissue samples from healthy controls and IL-1*β*, IL-8, and TNF-*α* were only measured at low concentrations (median concentrations; IL-1*β* 2.6 pg mg^−1^ total protein, IL-8 0.2 pg mg^−1^ total protein, TNF-*α* 0.1 pg mg^−1^ total protein). However, cytokine protein concentrations were significantly elevated in the tumour tissue: IL-1*β* 136 pg mg^−1^ of total protein (IQR 41–425), *P*=0.007; IL-6 3 pg mg^−1^ (IQR 0–46), *P*<0.05; IL-8 56 pg mg^−1^ (IQR 23–159), *P*=0.007; TNF-*α* 7 pg mg^−1^ (IQR 1–26), *P*<0.05 (Figure 3B). Of note, IL-1*β* concentrations were found at appreciably higher concentrations compared with the other cytokines (10–100-fold increase).

There was no correlation between tissue cytokine mRNA concentrations and cytokine tissue protein concentrations; IL-1*β* (*P*=0.64, *r*=0.07; Spearman's rank), IL-6 (*P*=0.46, *r*=−0.1), IL-8 (*P*=0.55, *r*=0.09), TNF-*α* (*P*=0.90, *r*=0.02). Increased mRNA concentrations were not associated with elevated tissue cytokine protein concentrations.

**Figure 4:**
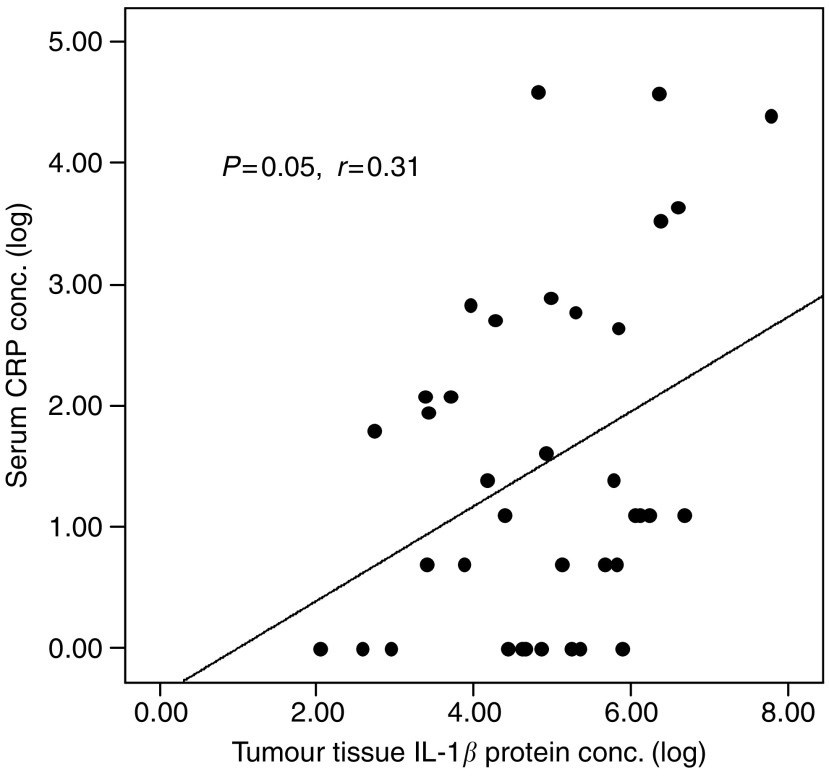
A scatter plot illustrating the relationship between serum CRP concentrations and tumour tissue IL-1*β* protein concentrations (*P*=0.05, *r*=0.31; linear regression).

Tissue cytokine mRNA concentrations did not correlate with serum cytokine concentrations or serum APP concentrations (data not shown). However, tumour tissue IL-1*β* protein levels were positively correlated with serum CRP concentrations (*P*=0.05, *r*=0.31; linear regression) (Figure 4). Although TNF-*α* protein levels did not correlate with serum cytokine/APP concentrations there was a significant correlation between sTNF-R and serum CRP concentrations (*P*=0.03, *r*=0.36). There was no correlation between tumour tissue IL-6 and either circulating IL-6 or APP concentrations. There was also a trend towards a correlation between tumour IL-8 protein concentrations and serum sTNF-R concentrations, but this did not quite reach statistical significance (*P*=0.06, *r*=0.32).

### Histological analysis

**Figure 5:**
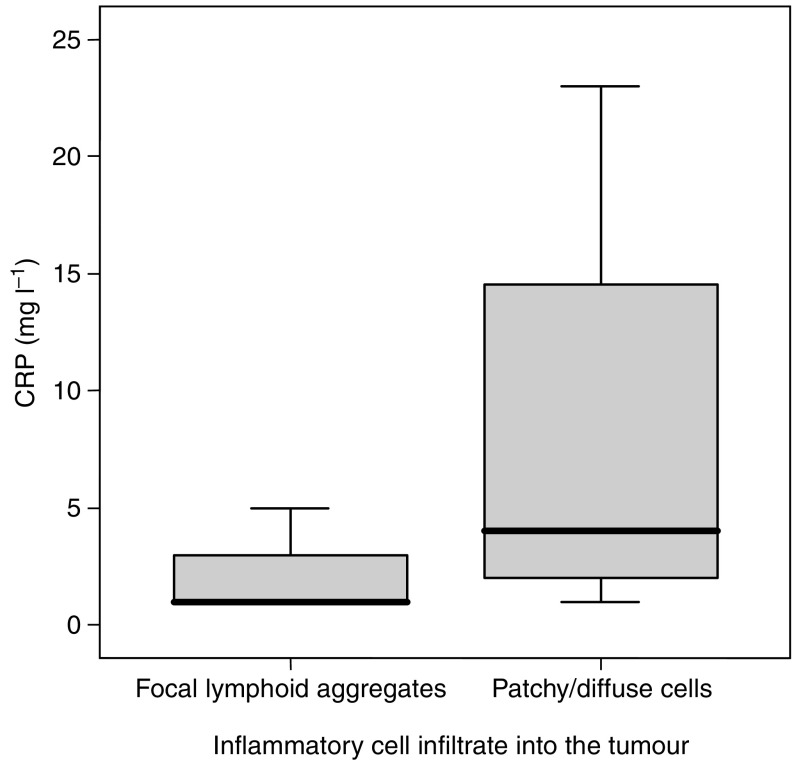
A diffuse or patchy inflammatory cellular infiltrate was associated with elevated serum CRP concentrations (*P*=0.01, Mann–Whitney *U*-test). Thick bar represents median, the box represents quartiles, and lines represent extreme values.

**Figure 6:**
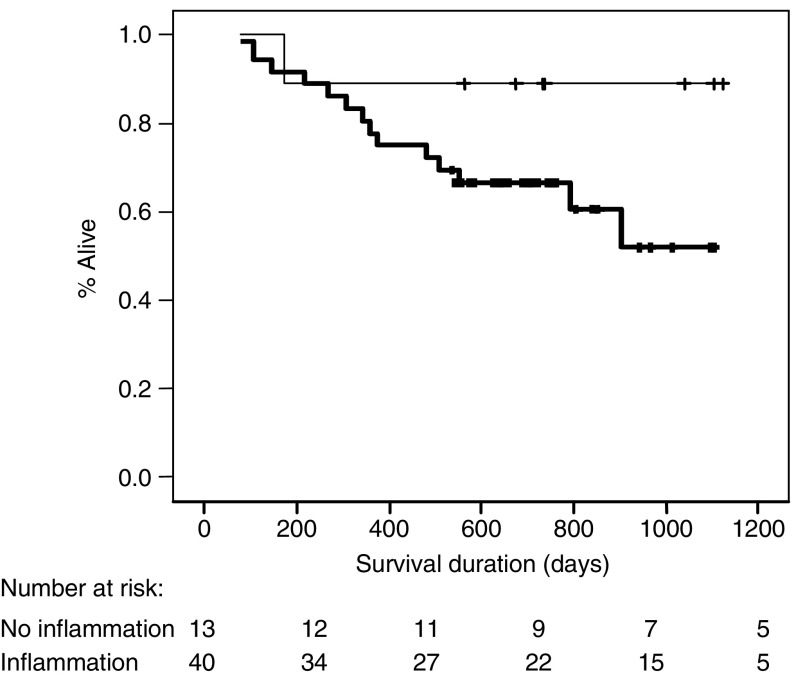
Kaplan–Meier survival plot presented by the presence or absence of a chronic inflammatory cellular infiltrate within the tumour. The heavy line represents the presence of a chronic inflammatory infiltrate *vs* focal lymphoid aggregates alone, light line (*P*=0.05; log-rank test).

Histology from three patients recruited to the study could not be traced; therefore, 53 tumour sections were studied. Twenty-four (45%) tumour samples were classified as having scanty diffuse or patchy chronic inflammatory cells. Sixteen (30%) tumour samples had a diffuse chronic inflammatory cellular infiltrate visible throughout the whole tumour. The remaining 13 (25%) tumour sections had focal lymphoid aggregates only. When compared with tumour sections possessing lymphoid aggregates alone, tissues with a diffuse or patchy inflammatory cellular infiltrate were associated with elevated serum CRP and sTNF-R concentrations (*P*=0.01 and *P*=0.007, respectively, Mann–Whitney *U*-test) (Figure 5). In addition, a chronic inflammatory cellular response was associated with reduced prognosis (*P*=0.05, log-rank test) (Figure 6). A chronic inflammatory infiltrate remained an independent prognostic indicator on multivariate analysis when analysed with stage, age, sex, tumour grade, and serum CRP concentrations (*P*=0.013, hazard ratio 7.7 (1.5–38.0 95% CI); Cox's proportional hazards model).

There was no correlation between the presence of a chronic inflammatory cell infiltrate and serum cytokine concentrations.

Six (11%) patients had histological evidence of *Helicobacter pylori* on the resected specimens. A chronic inflammatory cellular infiltrate was not associated with *H*. *pylori* infection (*P*=0.67, Fisher's exact test).

Tumour necrosis was evident in 13 (25%) samples, including four samples collected from patients who received preoperative chemotherapy, but there was no association between treatment modality and the presence of tumour necrosis (*P*=0.92, *χ*^2^ test). The presence of tumour necrosis was associated with elevated serum haptoglobin but not CRP concentrations (*P*=0.045 and *P*=0.07, respectively, Mann–Whitney *U*-test). Tumour necrosis was not associated with differences in tissue cytokine concentrations or survival (*P*=0.62, log-rank test).

Tissue cytokine (IL-1*β*, IL-6, IL-8, and TNF-*α*) mRNA and protein levels were found at similar concentrations within tumour tissues with a chronic inflammatory cell infiltrate and tumour samples with lymphoid aggregates alone.

## DISCUSSION

In this study, we have shown that patients with gastro-oesophageal malignancy have elevated serum concentrations of APPs but similar serum proinflammatory cytokine concentrations compared with a control population. A range of proinflammatory cytokine concentrations (mRNA and protein) were significantly elevated in tumour tissue compared with tissue sampled from healthy controls. However, only IL-1*β* correlated with markers of systemic inflammation (CRP). In addition, a chronic inflammatory cellular infiltrate within the tumour was associated with elevated serum APP concentrations and reduced survival, but was not associated with elevated tissue cytokine mRNA and protein concentrations.

An APPR has been well documented among patients with cancer, including gastric and oesophageal malignancies, and an elevated serum CRP has been identified as an adverse prognostic indicator, independent of stage of disease, among these patients (Rashid *et al*, 1982; Falconer *et al*, 1995; Kodama *et al*, 1999; McMillan *et al*, 2001, 2003; Nozoe *et al*, 2001; Forrest *et al*, 2003). The present study has confirmed these findings. Patients with gastro-oesophageal cancer had significantly elevated serum concentrations of positive APPs compared with healthy controls. Moreover, the 10 (18%) patients with a CRP concentration above 10 mg l^−1^ at diagnosis had a reduced survival interval, which was independent of disease stage. Our study did not demonstrate any differences in serum cytokine concentrations between cancer patients and controls. Although some studies have shown an association between serum cytokines and APPs (Martignoni *et al*, 2005) several have failed to demonstrate such a link and determination of serum cytokines remains an unreliable measure of tissue cytokine activity (Falconer *et al*, 1994; Barber *et al*, 1999). Moreover, these findings suggest that circulating cytokines may not be the key mediators of the APPR.

Proinflammatory cytokine mRNA and protein concentrations were either not detectable or found at low levels in tissue collected from healthy controls. In contrast, mRNA and cytokine protein concentrations were measured at significantly higher concentrations in tumour tissue. In all instances, tissue cytokine concentrations were significantly elevated in tumour tissue compared with tissue from healthy controls. These findings are supported by Yuan *et al* (2000) who investigated IL-8 mRNA concentrations in tumour tissue and adjacent normal lung tissue among patients with non-small-cell lung cancer and also found increased cytokine expression within the tumour tissue. Other groups have similarly demonstrated increased tissue cytokine concentrations associated with progression along the metaplasia–dysplasia–carcinoma sequence in Barrett's oesophagus (Tselepis *et al*, 2002; Dvorakova *et al*, 2004).

In the present study, median IL-1*β* concentrations were 10–100-fold higher than IL-6 in the tumour tissue and there was a weak but significant correlation between tumour tissue IL-1*β* concentration and serum CRP. There was a similar trend with IL-8. Both IL-1*β* and IL-8 are recognised as important cytokines in the generation of the systemic inflammatory response and it is possible that high tissue concentrations of these cytokines stimulate PBMCs as they pass through the tumour mass, which in turn act on target organs, such as the liver, to induce the synthesis of APPs that are associated with systemic inflammation. Previously, we have demonstrated that PBMC from weight-losing pancreatic cancer patients control the hepatic APPR by a primarily IL-6-dependent mechanism (O'Riordain *et al*, 1999). Moreover, Martignoni *et al* (2005) have suggested that IL-6 overexpression in cachectic pancreatic cancer patients is related to the ability of certain IL-6 producing tumours to sensitise PBMC and induce IL-6 expression in PBMCs. In the latter study, screening by DNA microassay analysis followed by quantitative PCR identified only IL-6 mRNA expression to be significantly increased in tumour samples of cachectic patients compared with noncachectic patients or pancreas samples from normal controls. Immunohistochemistry suggested the source of IL-6 to be tumour cells rather than host cells. The results of the present study, however, identify that at least in patients with gastro-oesophageal cancer IL-1*β* rather than IL-6 may be important as an initiator of the proinflammatory APPR. Interleukin-6 may form a common final pathway via activated PBMCs. Interestingly, in the colon-26 murine model of cancer cachexia associated with systemic inflammation there appears to be a complex intratumoural amplification loop between IL-1*β* and IL-6, which can be downregulated by IL-10 (Yasumoto *et al*, 1995; Fujiki *et al*, 1997).

In this study, we did not find any correlation between tissue cytokine mRNA concentrations and systemic cytokines or APP concentrations. Raddatz *et al* (2005) did identify an association between tissue cytokine mRNA levels and systemic CRP concentrations in Crohn's disease. These differing results may be partly explained by the lack of correlation between tissue mRNA concentrations and protein concentrations in this study. Although some groups have demonstrated a correlation between IL-1*β* and IL-6 mRNA and protein concentrations in an animal model of inflammatory joint disease, they also failed to show any correlation for TNF-*α* mRNA and protein concentrations (Rioja *et al*, 2004). The difficulties of relating mRNA concentrations to protein concentrations has been extensively documented elsewhere, but it is also important to consider that real-time PCR is an exquisitely sensitive technique and that what we are detecting in some patients, although elevated, may have little or no functional significance as it may not be translated into protein. Cytokine protein concentrations are, therefore, likely to be a more robust measure of tissue cytokine activity than mRNA levels.

A chronic inflammatory cellular infiltrate was noted in 40 (75%) tumour samples and was associated with elevated levels of serum CRP and sTNF-R. In addition, a chronic inflammatory infiltrate was associated with reduced survival. The presence of an inflammatory infiltrate within tumours and its relevance to prognosis has been investigated in a number of cancer types. Tumour-associated macrophages have been associated with reduced disease-free survival among lung, head and neck, and endometrial cancer (Marcus *et al*, 2004; Ohno *et al*, 2004; Chen *et al*, 2005). In contrast, increased numbers of tumour-associated macrophages, eosinophils, mast cells, and lymphocytes have been linked with improved survival in colorectal cancer (Svennevig *et al*, 1984; Jass, 1986; Nielsen *et al*, 1999). The prognostic significance of tumour-associated inflammatory cells is less clear in gastro-oesophageal cancer. An increased macrophage infiltrate was associated with more advanced stage of disease among patients with gastric cancer in one study, whereas other studies have suggested a more favourable prognosis associated with a more pronounced macrophage infiltration (Heidl *et al*, 1987; Tsujitani *et al*, 1987; Ohno *et al*, 2003). Similarly, increasing tumour-infiltrating lymphocyte count has been linked with decreased risk of death from gastric cancer in one study, but associated with an adverse prognosis in another (Setala *et al*, 1996; Grogg *et al*, 2003). Studies relating to oesophageal cancer are equally contradictory (Ma *et al*, 1999; Koide *et al*, 2004). In this study, there were no differences in tissue cytokine concentrations (mRNA or protein) between tumours with a chronic inflammatory infiltrate and those without, suggesting that differential tissue IL-1*β* expression is likely to be tumour-cell derived.

Laser capture microdissection (LCM) enables single cell types to be separated from multiple cell populations and would have been helpful in separating our tissue samples into pure tumour cell and inflammatory cell populations (Emmert-Buck *et al*, 1996). This technique was attempted initially but abandoned owing to inconsistent results, which were related to poor RNA quality as a consequence of this technique. In addition, our results have shown a lack of correlation between mRNA levels and functional protein concentrations, questioning the relevance of measuring mRNA concentrations. Determining cellular cytokine protein concentrations by the cytometric bead array system following LCM was not possible owing to the low protein concentrations that were retrieved.

Tumour necrosis was evident in 25% of tissue samples and was not associated with receipt of preoperative chemotherapy. The presence of tissue necrosis was weakly associated with elevated serum APP concentrations and may be explained by the necrotic tissue behaving like an abscess and inducing a predominantly acute inflammatory response. Tissue necrosis did not have any prognostic value in this study.

In conclusion, systemic inflammation is associated with adverse prognosis in gastro-oesophageal cancer. Tumour tissue cytokine concentrations are elevated compared with healthy controls and IL-1*β* concentrations are positively associated with some markers of systemic inflammation. In addition, the presence of a chronic inflammatory cell infiltrate into the tumour is also associated with markers of systemic inflammation and reduced survival, but is not associated with differential expression of tissue preinflammatory cytokine concentrations. This raises the possibility that the role of the chronic inflammatory infiltrate in the generation of systemic inflammation may be independent of differential expression of proinflammatory cytokines by these cells. Different mediators or cell–cell interactions may be more important for their effects.

## Figures and Tables

**Table 1:** Study patient demographics (*n*=56)

	**Number (%)**
Age (years)^a^	66 (58–75)
*Sex*	
Male	40 (71)
Female	16 (29)
	
*Tumour site*	
Oesophageal	26 (46)
Oesophago–gastric junction	13 (23)
Gastric	17 (30)
	
*Histology*	
Adenocarcinoma	52 (93)
Squamous cell carcinoma	4 (7)
	
*Grade*	
Well differentiated	4 (7)
Moderately differentiated	24 (43)
Poorly differentiated	28 (50)
	
*UICC stage*	
1	17 (30)
2	13 (23)
3	21 (38)
4	5 (9)
	
*Treatment undertaken*	
Oesophagectomy	25 (45)
Gastrectomy	18 (32)
Preoperative chemotherapy followed by surgery	13 (23)
	
*Status*	
Alive	37 (66)
Dead	19 (34)

a Values given are median (interquartile range).

**Table 2:** Serum concentrations of acute phase proteins and cytokines for the patient group (*n*=56) and healthy controls (*n*=22)

	**Patient group (*n*=56)**	**Control group (*n*=22)**	***P*-value^a^**
CRP (mg/l)	4 (2–16)	1 (1–3)	<0.001
Haptoglobin (mg/l)	1869 (1421–2651)	821 (627–1157)	<0.001
ACT (mg/l)	409 (326–502)	245 (213–261)	<0.001
Albumin (g/l)	42 (39–44)	42 (39–45)	0.227
Transferrin (mg/l)	2076 (1565–2648)	2197 (1861–2451)	0.478
IL-1*β* (pg/ml)	0^b^	0	—
IL-6 (pg/ml)	0 (0–91)	11 (0–214)	0.412
IL-8 (pg/ml)	0 (0–57)	0 (0–118)	0.683
IL-10 (pg/ml)	0^c^	0	—
sTNF-R (ng/ml)	2.6 (1.3–4.1)	2.8 (1.3–3.6)	0.559

ACT=*α*1-antichymotrypsin; CRP=C-reactive protein; IL=interleukin; sTNF-R=soluble tumour necrosis factor receptor (p55).

Positive acute phase protein concentrations were elevated in the patient group compared with the control group. There were no differences between concentrations of the negative acute phase reactants or serum cytokines.

a Mann–Whitney *U*-test.

b Only two patients had measurable serum IL-1*β* concentrations.

c Only four patients had measurable IL-10 concentrations. Values are median (interquartile range).
